# Consensus-Based Track Association with Multistatic Sensors under a Nested Probabilistic-Numerical Linguistic Environment

**DOI:** 10.3390/s19061381

**Published:** 2019-03-20

**Authors:** Xinxin Wang, Zeshui Xu, Xunjie Gou

**Affiliations:** 1 Business School, State Key Laboratory of Hydraulics and Mountain River Engineering, Sichuan University, Chengdu 610064, China; wangxinxin_cd@163.com (X.W.); gou_xunjie@163.com (X.G.); 2 School of Computer and Software, Nanjing University of Information Science & Technology, Nanjing 210044, China

**Keywords:** track association, multi-static sensors, MAGDM, NPNLTSs, consensus model

## Abstract

Track association is an important technology in military and civilian fields. Due to the increasingly complex environment and the diversity of the sensors, it is a key factor to separate the corresponding track from multiple maneuvering targets by multisensors with a consensus. In this paper, we first transform the track association problem to multiattribute group decision making (MAGDM), and describe the MAGDM with nested probabilistic-numerical linguistic term sets (NPNLTSs). Then, a consensus model with NPNLTSs is constructed which has two key processes. One is a consensus checking process, and the other is a consensus modifying process. Based on which, a track association algorithm with automatic modification is put forward based on the consensus model. After that, the solution of a case study in practice is given to obtain the corresponding track by the proposed method, and it provides technical support for the track association problems. Finally, we make comparisons with other methods from three aspects, and the results show that the proposed method is effective, feasible, and applicable. Moreover, some discussions about the situation where there is only one echo point at a time are provided, and we give a discriminant analysis method.

## 1. Introduction

Track association has become an indispensable technology in modern information systems, and has made a great contribution to military and civil applications [1,2]. The aim of track association problems is to separate tracks from multiple maneuvering targets by sensors. Since sensors have their errors of key parameters, such as distance, azimuth angle, and pitch angle, and each sensor has its own assessment result, it is very important to obtain the effective and accurate extraction of useful information with a consensus. Due to the complex and uncertain environment, the challenges of the track association process are as follows: (1) difficulty in describing the information of each target point accurately considering some systematic errors of the sensor and outside interference [3]; (2) obtaining the corresponding track from targets with a higher consensus by a multisensor [4,5]. The reason is that compared with the single sensor, multisensor systems are shown to be more accurate and reliable, and the most important problem is the data association with a consensus. Because of the described challenges and the tactical advantages, track association for targets by multisensors with a consensus has become an interesting and difficult research topic [6,7,8].

Over the years, on the one hand, some approaches and algorithms have been developed for track association, such as the Gaussian mixture model (GMM) [9], multiple hypothesis tracking (MHT) [10], and joint probabilistic data association (JPDA) [11,12]. Moreover, fuzzy algorithm [13,14], Dempster–Shafer (D–S) evidence theory [15], and neural network [16] were also introduced to handle track association problem. Specifically, the GMM is applied to represent and filter the uncertainty in track association caused by error of the sensors. Although this approach robustly tracks the trajectory of a target, the target problem stemming from insufficient measurements may still cause performance degradation, and the other problem is how to reach a consensus among multisensors. MHT and JPDA can deal with the consensus problem and improve the performance for track association. However, they suffer from a heavy computational load when highly cluttered environments occur, and the track often fails to achieve accurate target tracking under such circumstances. On the other hand, some state-of-the-art consensus-based methods for tracking and positioning have also been developed. For example, the consensus Monto Carlo algorithm was proposed to process comfortably on a single machine [17]. A linear fusion rules method was put out based on both a constrained optimization perspective and a Bayesian approach [18]. The connections between consensus problem in networked dynamic systems and diverse applications were discussed [19]. Some issues related to gossiping over wireless links were studied, and the use of gossip algorithms for canonical signal processing tasks were illustrated including distributed estimation, source localization, and compression [20]. However, the above consensus-based methods cannot deal with track association problems efficiently under complex environments. Therefore, it is a challenge to obtain the accurate track and improve the reliability and the consensus among sensors under the complicated, uncertain, and varying circumstances [21].

Recently, the nested probabilistic-numerical linguistic term sets (NPNLTSs) [22], which has been used to deal with the decision and optimization problems, was applied to handle the maneuvering target tracking problem by multisensors [23]. To some extent, the track association problem can be considered as a multiattribute group decision making (MAGDM) problem. As a form of the information expression, NPNLTSs can describe evaluation information more accurately through the nested information, such as errors of sensors, in the track association problem. Considering that there are uncertainties regarding qualitative information and quantitative information in the process of track association, we can use NPNLTSs to describe the MAGDM problem. Compared with other popular linguistic sets applied to many fields [24,25,26], such as hesitant fuzzy linguistic term sets (HFLTSs) [27], probabilistic linguistic term sets (PLTSs) [28], and double hierarchy hesitant fuzzy linguistic term sets (DHHFLTSs) [29], NPNLTSs can provide a different and more powerful form to fully represent preference information, and the distance and similarity measures of NPNLTSs have been proposed [30]. In this paper, we use NPNLTSs to optimize the error of sensors, give evaluation information, and establish the consensus model with NPNLTSs. The key to deal with the problem is to set up an effective algorithm with the information as accurate as possible in the process of track association. For example, how to ensure the accuracy of the measurement data by sensors; how to make information integrity during the processing of the algorithm; how to distinguish abnormal points among the track points; and how to deal with the situation where there is only one echo point at a time.

Given the above-mentioned issues, this paper focuses on how to determine each track point belonging to which maneuvering target with a higher consensus in the track association problem. Its three main contributions are as follows: (1) considering qualitative information and quantitative information in complex evaluation environments, and errors information of key parameters, we describe uncertain information more accurately based on nested probabilistic-numerical linguistic information. (2) A consensus model with NPNLTSs is constructed which has two key processes. One is a consensus checking process, and the other is a consensus modifying process. The track association algorithm with automatic modification is put forward based on the consensus model. (3) A solution of a case study in practice is presented by the proposed method, which can provide technical support for the track association problems, and it is effective, feasible, and precise compared with other methods. Moreover, a situation when there is only one echo point at a time by sensors is discussed, and the discriminant analysis method is given.

The rest of this paper is organized as follows: Section 2 presents the assumptions and notations. In Section 3, we give the methodologies including MAGDM with NPNLTSs, the consensus model, and the track association algorithm. Section 4 deals with a case study by the proposed method. In Section 5, we make comparisons among other three methods from three aspects and discuss the situation where there is only one echo point at a time by sensors. Section 6 ends the paper with some conclusions.

## 2. Assumptions and Abbreviations

For convenience and simplicity of the future discussions, some basic assumptions and the main abbreviations are listed as follows:

### 2.1. Assumptioins

Since the track association problem has many uncertain factors under the complex environment, we need to make some assumptions to ensure the accuracy of the model.

Assumptions about parameters to assure the accuracy of coordinate calculation:

- The earth is a sphere with a radius of 6371 km.
- The gravitational acceleration is $g=10\;m/s^{2}$.
- The interference factors obey Gaussian white noise.

Assumptions about sensors to assure the reliability of data:

- Ignore the time of transmitting and receiving light waves of the sensor.
- The speed of light is infinite.
- The maneuvering target and the sensor are particles.

### 2.2. Abbreviations


**Variables**

**Descriptions**

$s_{q}$
q-th sensor, q=1,2,⋯,k
$t_{i}$
i-th track point, i=1,2,⋯,p
$c_{j}$
j-th attribute, j=1,2,⋯,m
$x_{l}$
l-th maneuvering target, l=1,2,⋯,n
D
Distance between target and the sensor 
α
Azimuth angle
β
Pitch angle
ξ
The consensus threshold
τ
The adjustment coefficient
$w_{q}$
q-th weight with respect to the sensor $s_{q}$, q=1,2,⋯,k
$\omega_{j}$
j-th weight with respect to the attribute $c_{j}$, j=1,2,⋯,m
**Acronyms**

**Full name**
MAGDMMulti-attribute group decision makingNPNLTSsNested probabilistic-numerical linguistic term setsGMMGaussian mixture modelMHTMultiple hypothesis trackingJPDAJoint probabilistic data associationHFLTSsHesitant fuzzy linguistic term setsPLTSsProbabilistic linguistic term setsDHHFLTSsDouble hierarchy hesitant fuzzy linguistic term sets

## 3. Methodology

In order to utilize detection data rationally by multistatic sensors and make track-association for multiple maneuvering targets accurately, we first introduce the MAGDM with NPNLTSs between the group sensors, with the aim of reaching an acceptable and precise decision result, and then establish a consensus model in the MAGDM problem. Furthermore, a track-association algorithm based on the consensus model is proposed.

### 3.1. MAGDM with NPNLTSs

In this subsection, we review some basic concepts of NPNLTSs in detail and describe the MAGDM problem of track association with NPNLTSs.

#### 3.1.1. NPNLTSs

NPNLTSs can express nested information in complex circumstances, and pioneering work has been performed on this topic [22,23]. In order to model decision-making situations adequately in which the decision makers describe qualitative and quantitative information simultaneously, the NPNLTSs was developed. The form of the NPNLTS is NPN={OL(p){IL(v)}}, which consists of an outer-layer probabilistic linguistic term set (OPLTS) OL(p) and an inner-layer numerical linguistic term set (INLTS) IL(v) denoted as [22]:

(1) $$OL\left(p\right)=\left\{OL^{\left(k\right)}\left(p^{\left(k\right)}\right)|OL^{\left(k\right)}\in OS,\;p^{\left(k\right)}\geq 0,\;k=1,2,\cdots ,\#OL\left(p\right),\sum_{k=1}^{\#OL\left(p\right)}p^{\left(k\right)}\leq 1\right\},$$

(2) $$IL\left(v\right)=\left\{IL_{\left(k\right)}^{\left(l\right)}\left(v_{\left(k\right)}^{\left(l\right)}\right)|IL_{\left(k\right)}^{\left(l\right)}\in IS,\;v_{\left(k\right)}^{\left(l\right)}\geq 0,\;k=1,2,\cdots ,\#OL\left(p\right),l=1,2,\cdots ,\#IL\left(v\right)\right\}.$$

where $OS=\{s_{\alpha }|\alpha =0,1,2,\cdots ,\tau \}$ and $IS=\{n_{\beta }|\beta =0,1,2,\cdots ,\varsigma \}$ are called an outer-layer linguistic term set (OLTS) and an inner-layer linguistic term set (ILTS), respectively, in the nested linguistic term set (NLTS) $NS=\{s_{\alpha }\left\{n_{\beta }\right\}\}$. $OL^{\left(k\right)}\left(p^{\left(k\right)}\right)$ is the *k*-th outer-layer linguistic term element (OLTE) in the OLTS associated with the probability $p^{\left(k\right)}$, and #OL(p) is the number of the linguistic term elements in OL(p). $IL_{\left(k\right)}^{\left(l\right)}\left(v_{\left(k\right)}^{\left(l\right)}\right)$ is the *l*-th inner-layer linguistic term element (ILTE) in the ILTS associated with the value $v_{\left(k\right)}^{\left(l\right)}$ under the *k*-th OLTE, and #IL(v) is the number of the linguistic term elements in IL(v). Moreover, the form of the normalized NPNLTS (N-NPNLTS) [22] is $\bar{NPN}=\left\{OL^{N}\left(p\right)\left\{IL^{N}\left(v\right)\right\}\right\}$ that consists of a normalized OPLTS $OL^{N}\left(p\right)$ and a normalized INLTS $IL^{N}\left(v\right)$:

(3) $$OL^{N}\left(p\right)=\left\{OL^{N}{}^{\left(k\right)}\left(p^{N}{}^{\left(k\right)}\right)|OL^{N}{}^{\left(k\right)}\in OS,\;p^{N}{}^{\left(k\right)}\geq 0,\;k=1,2,\cdots ,\tau +1,\sum_{k=1}^{\tau +1}p^{N}{}^{\left(k\right)}=1\right\},$$

(4) $$IL^{N}\left(v\right)=\left\{IL_{\left(k\right)}^{N\left(l\right)}\left(v_{\left(k\right)}^{N\left(l\right)}\right)|IL_{\left(k\right)}^{N\left(l\right)}\in IS,\;1\geq v_{\left(k\right)}^{N\left(l\right)}\geq 0,\;k=1,2,\cdots ,\tau +1,l=1,2,\cdots ,\varsigma +1\right\}.$$

where $p^{N(k)}=p^{\left(k\right)}/\sum_{k=1}^{\tau +1}p^{\left(k\right)}$ and $v_{\left(k\right)}^{N\left(l\right)}=v_{\left(k\right)}^{\left(l\right)}/\sum_{k=1}^{\tau +1}v_{\left(k\right)}^{\left(l\right)}$.

For example, let OS and IS be an OLTS and an ILTS in a NLTS, respectively, i.e.,

$$OS=\left\{s_{0}:poor\;student,s_{1}:average\;student,s_{2}:top\;student\right\}$$

$$IS=\left\{n_{0}:attention\;score,n_{1}:attitude\;score,\;n_{2}:intelligence\;score\right\}$$

Then, there are two N-NPNLTSs as follows:

$${\bar{NPN}}_{1}=\left\{\begin{array}{l} s_{0}(0.3)\left\{n_{0}\left(0.7\right),n_{1}\left(0.5\right),n_{2}\left(0.8\right)\right\},\\ s_{1}(0.4)\left\{n_{0}\left(0.8\right),n_{1}\left(0.6\right),n_{2}\left(0.8\right)\right\},\\ s_{2}(0.3)\left\{n_{0}\left(0.9\right),n_{1}\left(0.7\right),n_{2}\left(0.7\right)\right\} \end{array}\right\},\;{\bar{NPN}}_{2}=\left\{\begin{array}{l} s_{0}(0.6)\left\{n_{0}\left(0.7\right),n_{1}\left(0.5\right),n_{2}\left(0.8\right)\right\},\\ s_{1}(0.4)\left\{n_{0}\left(0.8\right),n_{1}\left(0.6\right),n_{2}\left(0.8\right)\right\} \end{array}\right\}$$

where $OL_{1}^{N}\left(p\right)$ and $OL_{2}^{N}\left(p\right)$ are $OL_{1}^{N}\left(p\right)=\left\{s_{0}(0.3),s_{1}(0.4),s_{2}(0.3)\right\}$, $OL_{2}^{N}\left(p\right)=\left\{s_{0}(0.6),s_{1}(0.4)\right\}$ and $IL^{N}\left(v\right)=\left\{\left\{n_{0}\left(0.7\right),n_{1}\left(0.5\right),n_{2}\left(0.8\right)\right\},\left\{n_{0}\left(0.8\right),n_{1}\left(0.6\right),n_{2}\left(0.8\right)\right\},\left\{n_{0}\left(0.9\right),n_{1}\left(0.7\right),n_{2}\left(0.7\right)\right\}\right\}$.

Here, ${\bar{NPN}}_{1}$ means that after the assessment for a student, the probabilities of the poor student, average student, and top student are 0.3, 0.4, and 0.3, respectively. The sum of the probabilities is 1. Meanwhile, if the student is a poor one, the attention score, attitude score, and intelligence score after normalization are 0.7, 0.5 and 0.8, respectively; if the student is an average student, they are 0.8, 0.6, and 0.8, respectively, and if the student is a top student, they are 0.9, 0.7, and 0.7, respectively. Specifically, the first element $s_{0}(0.3)\left\{n_{0}\left(0.7\right),n_{1}\left(0.5\right),n_{2}\left(0.8\right)\right\}$ in the ${\bar{NPN}}_{1}$ means that the probability of the poor student is 0.3, and the attention score, attitude score and intelligence score for him/her are 0.7, 0.5, and 0.8, respectively. Similarly, we can interpret other elements in ${\bar{NPN}}_{1}$, and the meaning for ${\bar{NPN}}_{2}$.

#### 3.1.2. MAGDM Problem

A track association MAGDM problem with NPNLTSs between the group sensors based on consensus can be described as follows:

Let $X=\left\{x_{1},x_{2},\cdots ,x_{n}\right\}\left(n\geq 2\right)$ be a set of maneuvering targets, $T=\left\{t_{1},t_{2},\cdots ,t_{p}\right\}\left(p\geq 2\right)$ be a set of track points measured by a set of sensors $S=\left\{s_{1},s_{2},\cdots ,s_{k}\right\}\left(k\geq 2\right)$, where $E^{k}=\left\{e_{1}^{k},e_{2}^{k},\cdots ,e_{r}^{k}\right\}\left(r\geq 2\right)$ is a set of systematic errors of a sensor $s_{k}$ and $w={\left(w_{1},w_{2},\cdots ,w_{k}\right)}^{T}$ is the associated weight vectors over sensors S, with $0\leq w_{k}\leq 1$ and $\sum_{q=1}^{k}w_{q}=1\;\left(q=1,2,\cdots ,k\right)$. Let $OS=\left\{s_{0}:x_{1},s_{1}:x_{2},\cdots ,s_{n-1}:x_{n}\right\}$ be the OLTS, $IS=\left\{n_{0}:e_{1},n_{1}:e_{2},\cdots ,n_{r-1}:e_{r}\right\}$ be the ILTS, and $C=\left\{c_{1},c_{2},\cdots ,c_{m}\right\}\left(m\geq 2\right)$ be several attributes with a weighting vector $\omega ={\left(\omega_{1},\omega_{2},\cdots ,\omega_{m}\right)}^{T}$, where $\omega_{j}\geq 0\left(j=1,2,\cdots ,m\right)$ denotes the weight of attribute $c_{j}$ and $\sum_{j=1}^{m}\omega_{j}=1$. Let $NPN^{q}={\left(NPN_{ij}^{q}\right)}_{p\times m}\left(q=1,2,\cdots ,k\right)$ be the decision matrix given by sensor $s_{q}\in S$, where $NPN_{ij}^{q}$ represents the evaluation information for the track point $t_{i}\in T$ with respect to the attribute $c_{j}\in C$.

The key to the track association problem between the group sensors is how to determine each track point belonging to which maneuvering target with a higher consensus. At present, solving the MAGDM problem with NPNLTSs mainly concerns three phases: (1) normalization phases; (2) aggregation phases; (3) decision phase.

(1) Normalization phases

In general, there are benefit attributes and cost attributes in traditional MAGDM problem. However, in the track association problem, the expression of information is NPNLTSs and the ILTS is about errors. Therefore, the attributes are all considered as cost attributes.

(2) Aggregation phases

In order to aggregate each individual evaluation matrix by each sensor into a collective one and further aggregate all the attributes of the collective evaluation matrix, an aggregation operator is needed, such as the weighted average (WA) operator and the ordered weighted average (OWA) operator. Without loss of generality, we use the WA operator to aggregate individual evaluation matrix and all the attributes.

Let $w={\left(w_{1},w_{2},\cdots ,w_{k}\right)}^{T}$ and $NPN^{q}={\left(NPN_{ij}^{q}\right)}_{p\times m}\left(q=1,2,\cdots ,k\right)$ be defined as before. Then, we can obtain a collective evaluation matrix $NPN^{c}=\left(NPN_{ij}^{c}\right)$ using the WA operator, where

(5) $$NPN_{ij}^{c}=\sum_{q=1}^{k}w_{q}\cdot NPN_{ij}^{q}.$$

Let $\omega ={\left(\omega_{1},\omega_{2},\cdots ,\omega_{m}\right)}^{T}$ be defined as before, and $z={\left(z_{1},z_{2},\cdots ,z_{p}\right)}^{T}$ be the overall evaluation vector over track point T, where $z_{i}\left(i=1,2,\cdots ,p\right)$ is the overall evaluation value of track point $t_{i}$ derived from the collective evaluation matrix $NPN^{c}={\left(NPN_{ij}^{c}\right)}_{p\times m}$. Similarly, $z_{i}$ can be obtained using WA operator, i.e.,

(6) $$z_{i}=\sum_{j=1}^{m}\omega_{j}\cdot NPN_{ij}^{c}.$$

(3) Decision phases

Based on $z_{i}\left(i=1,2,\cdots ,p\right)$, considering the various systematic errors of each sensor, the most likely maneuvering target for each track point $t_{i}$ can be obtained.

### 3.2. Consensus Model in NPNLTSs

Since existing research about the track association MAGDM problem with NPNLTSs cannot guarantee the consensus among sensors, we establish a consensus model with NPNLTSs in MAGDM to achieve a collective solution with a consensus, and there are two key processes: the consensus checking process and consensus modifying process.

#### 3.2.1. Consensus Checking Process

In consensus-based track association MAGDM problem, consensus measure shows the distance between the evaluation information of sensors. Let $z^{q}={\left(z_{1}^{q},z_{2}^{q},\cdots ,z_{p}^{q}\right)}^{T}\left(q=1,2,\cdots ,k\right)$ be the individual overall evaluation vectors derived from $NPN^{q}={\left(NPN_{ij}^{q}\right)}_{p\times m}$, and $z^{c}={\left(z_{1}^{c},z_{2}^{c},\cdots ,z_{p}^{c}\right)}^{T}$ be the collective overall evaluation vector over track points derived from $NPN^{c}={\left(NPN_{ij}^{c}\right)}_{p\times m}$ using Equation (6). The consensus degree among sensors $S=\left\{s_{1},s_{2},\cdots ,s_{k}\right\}\left(k\geq 2\right)$ is calculated by

(7) $$CD\left(s_{1},s_{2},\cdots ,s_{k}\right)=\frac{1}{k\cdot p}\sum_{q=1}^{k}\sum_{i=1}^{p}\left|z_{i}^{q}-z_{i}^{c}\right|.$$

Obviously, $CD\left(s_{1},s_{2},\cdots ,s_{k}\right)\in \left[0,1\right]$. Specifically, if $CD\left(s_{1},s_{2},\cdots ,s_{T}\right)=0$, then all sensors are at full consensus with the collective evaluation information. Otherwise, the smaller value of $CD\left(s_{1},s_{2},\cdots ,s_{k}\right)$, the higher consensus level among all sensors. Based on the ranges of correlation strength [31], the strength of consensus degree can be defined as follows:

(1) $CD\left(s_{1},s_{2},\cdots ,s_{k}\right)\in \left[0,0.2\right]$ indicates extremely strong consensus;
(2) $CD\left(s_{1},s_{2},\cdots ,s_{k}\right)\in \left(0.2,0.4\right]$ indicates strong consensus;
(3) $CD\left(s_{1},s_{2},\cdots ,s_{k}\right)\in \left(0.4,0.6\right]$ indicates moderate degree consensus;
(4) $CD\left(s_{1},s_{2},\cdots ,s_{k}\right)\in \left(0.6,0.8\right]$ indicates weak consensus;
(5) $CD\left(s_{1},s_{2},\cdots ,s_{k}\right)\in (0.8,1]$ indicates extremely weak consensus or no consensus.

#### 3.2.2. Consensus Modifying Process

When consensus between group sensors is low after the consensus checking process, the consensus modifying process is needed to achieve a higher consensus level. Traditionally, the consensus modifying process is based on two classical rules [32,33,34]; the identification rule and direction rule. Automatic modification is low cost and has been applied to many fields [35,36]. Let ξ be the given consensus threshold. If the consensus degree $CD\left(s_{1},s_{2},\cdots ,s_{k}\right)>\xi$, then the individual evaluation vectors $NPN_{ij}^{q}$ should be adjusted as ${\tilde{NPN}}_{1ij}^{q}$ with an adjustment coefficient τ, where

(8) $${\tilde{NPN}}_{1ij}^{q}=\frac{1}{\tau }\left(NPN_{ij}^{q}+NPN_{ij}^{c}\right).$$

If the consensus degree $CD\left(s_{1},s_{2},\cdots ,s_{k}\right)$ is smaller than the given consensus threshold ξ, then the final group evaluation value $z_{i}\left(i=1,2,\cdots ,p\right)$ can be calculated by aggregating all the individual evaluation vectors using Equation (6).

Combined with the strength of consensus degree, the value of the consensus threshold ξ can be determined as ξ∈[0.4,0.8]. Without loss of generality, in this paper, the adjustment coefficient τ is 1/2. Moreover, the consensus threshold ξ and the adjustment coefficient τ can be adjusted according to the practical situation of the MAGDM problem. Specifically, if the demand for consensus is strict and the requirement for speed is rapid, the consensus threshold ξ and the adjustment coefficient τ should both be given lower values; otherwise, higher values of them should be provided.

### 3.3. Track Association Algoritm

**Figure 1:**
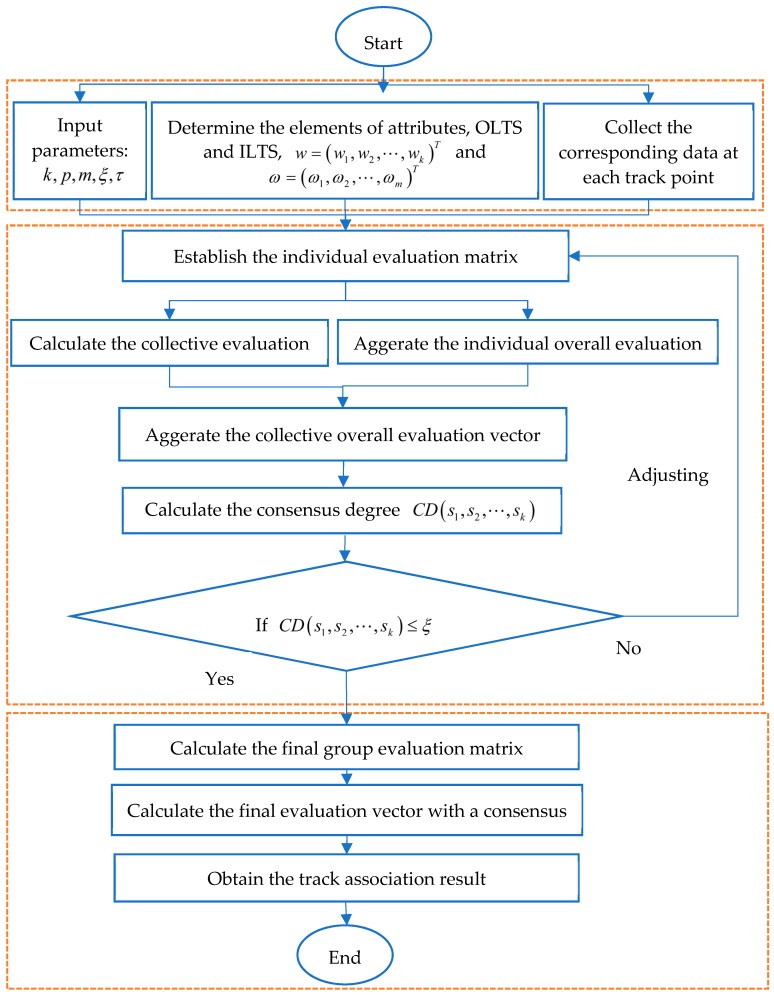
The flowchart of the track association algorithm based on the consensus model with nested probabilistic-numerical linguistic term sets (NPNLTSs).

In the following, we propose a track association algorithm to help sensors achieve a consensus and determine each track point belonging to each maneuvering target. The key is that sensors modify their evaluation information based on the individual overall evaluation information and the collective overall evaluation information. In order to clarify the proposed consensus model with NPNLTSs, we summarize the procedure as Algorithm 1 and further illustrate the algorithm by a flowchart (Figure 1).

**Algorithm 1.** The track association algorithm based on consensus model with NPNLTSs.
**Step 1.**
(1) Input the parameters: k,p,m,ξ,τ. (2) Determine the attributes, OLTS, ILTS and the weight vectors $w={\left(w_{1},w_{2},\cdots ,w_{k}\right)}^{T}$ and $\omega ={\left(\omega_{1},\omega_{2},\cdots ,\omega_{m}\right)}^{T}$. (3) Collect the corresponding data at each track point $T=\left\{t_{1},t_{2},\cdots ,t_{p}\right\}\left(p\geq 2\right)$ measured by a set of sensors $S=\left\{s_{1},s_{2},\cdots ,s_{k}\right\}\left(k\geq 2\right)$. Go to the next step.
**Step 2.**
Based on the OLTS and the ILTS, establish the individual evaluation matrix $NPN^{q}={\left(NPN_{ij}^{q}\right)}_{p\times m}$ with the sensor $s_{q}\in S\left(q=1,2,\cdots ,k\right)$ for the track point $t_{i}\in T$ with respect to the attribute $c_{j}\in C$. Go to the next step.
**Step 3.**
(1) Calculate the collective evaluation matrix $NPN^{c}=\left(NPN_{ij}^{c}\right)$ using Equation (5). $\mathrm{Go}\;\mathrm{to}\;\mathrm{the}\;\mathrm{next}\;\mathrm{step}.\;(2)\;\mathrm{Aggregate}\;\mathrm{the}\;\mathrm{individual}\;\mathrm{overall}\;\mathrm{evaluation}\;\mathrm{vectors}\;z^{q}={\left(z_{1}^{q},z_{2}^{q},\cdots ,z_{p}^{q}\right)}^{T}$ using Equation (6), with the associated weight vector $w={\left(w_{1},w_{2},\cdots ,w_{k}\right)}^{T}$ over sensors S. Go to the next step.
**Step 4.**
Aggregate the collective overall evaluation vector $z^{c}=\left(z_{1}^{c},z_{2}^{c},\cdots ,z_{p}^{c}\right)$ using Equation (6), with the associated weight vector $\omega ={\left(\omega_{1},\omega_{2},\cdots ,\omega_{m}\right)}^{T}$ over attributes C. Go to the next step.
**Step 5.**
Determine the consensus threshold ξ, which is in the range of [0.4, 0.8] generally, and the adjustment coefficient τ, in this paper, we let τ=1/2. Go to the next step.
**Step 6.**
Calculate the consensus degree $CD\left(s_{1},s_{2},\cdots ,s_{k}\right)$ by Equation (7). If $CD\left(s_{1},s_{2},\cdots ,s_{k}\right)>\xi$, then go to the next step; Otherwise, go to Step 8.
**Step 7.**
Adjust the individual evaluation matrix $NPN^{q}={\left(NPN_{ij}^{q}\right)}_{p\times m}\left(q=1,2,\cdots ,k\right)$ by Equation (8) until $CD\left(s_{1},s_{2},\cdots ,s_{k}\right)\leq \xi$. Go to the next step.
**Step 8.**
Aggregate all the individual evaluation matrices into a final group evaluation matrix $NPN={\left(NPN_{ij}\right)}_{p\times m}$ by Equation (5). Go to the next step.
**Step 9.**
Obtain the most likely maneuvering target at each track point $t_{i}\in T$ based on Equation (6). Go to the next step.
**Step 10.**
End.

As we can see in Figure 1, the track association algorithm based on the consensus model has three stages, i.e., preparation stage, calculation stage, and decision stage. Specifically, we mainly input parameters, determine weight vectors, and collect data in the preparation stage. Then, we calculate the corresponding matrices, vectors, and consensus degree in the calculation stage. Finally, we calculate the final evaluation vector with a consensus and make a decision in the late stage.

In the following, we give the pseudo-code of the algorithm as follows:

**Pseudo-code**. The pseudo-code of the track association algorithm.Input parameters: *k*—the number of sensors;  *p*—the number of track points; *m*—the number of attributes; ξ—the consensus threshold; τ—the adjustment coefficient; w—the weight of the sensors;  ω—the weight of the attributes. 1. // Calculate the collective evaluation matrix 2. for *i*: = 1 to *p* 3.   for *j*: = 1 to *k* 4.   collective. element (*i*, *j*): = sum (sensor (*i*, *j*) * w(*j*)) 5. // Calculate the consensus degree 6. for *i*: = 1 to *p* 7.   for *j*: = 1 to *m* 8.   individual. element (*i*, *j*): = sum (attribute (*i*, *j*) * ω(*j*)) 9.   overall. element (*i*): = sum (individual. element (*i*, *j*) * ω(*j*)) 10.    consensus = sum (abs (individual. element (*i*)—overall. element (*i*)))/*p* 11. // Calculate the final result with a consensus 12. while (consensus < ξ) 13.    ad_individual. element (*i*, *j*): = (individual. element (*i*, *j*) + collective. element (*i*, *j*))/τ 14.    group. element (*i*, *j*): = ad_individual. element (*i*, *j*) * w(*j*)) 15.    final. element (*i*): = group. element (*i*, *j*) *ω(*j*)16.    return max_final. element (*i*)

## 4. A Case Study

Multiple target tracking is very important to predict the intentions or motive of the maneuvering targets in military, transportation, and navigation-related fields. Maneuvering target tracking technology with multiple sensors is indispensable in securing information assurance, financial security, and personal safety. In this section, we deal with a track association problem of multiple maneuvering targets with multistatic sensors by the proposed method.

### 4.1. Problem Description

In a military drill, two maneuvering targets are both required to make flight verification test throughout the flight envelope. In general, a flight verification test involves the army setting a specific flight path according to the national military standard rules to evaluate the performance of the sensor. Therefore, the flight envelope is relevant to actual combat. In order to obtain the corresponding track, there are three sensors that record flight parameters of the two targets at different times, and the relevant data are returned from real-world experiments in real time. Part of the measurement data is recorded in Table 1.

It is noted that each sensor records 883 sets of measurement data, and there is a total of 2649 sets of measurement data measured by various sensors. Moreover, there is no identical recording time among the three sensors. The correlation system information about the three sensors is shown in Table 2.

As we can see in Table 2, the three sensors are located in the horizontal plane, which can be inferred by their heights, and they are all located at an eastern longitude and northern latitudes. Additionally, the sensors’ systemic error parameters in Table 2 are the maximum errors considering multiple factors in as many cases as possible during the tracking process. Combined with the information above, the goal is to obtain the tracks of two maneuvering targets precisely with a consensus.

### 4.2. Establishing the Proposed Model

**Figure 2:**
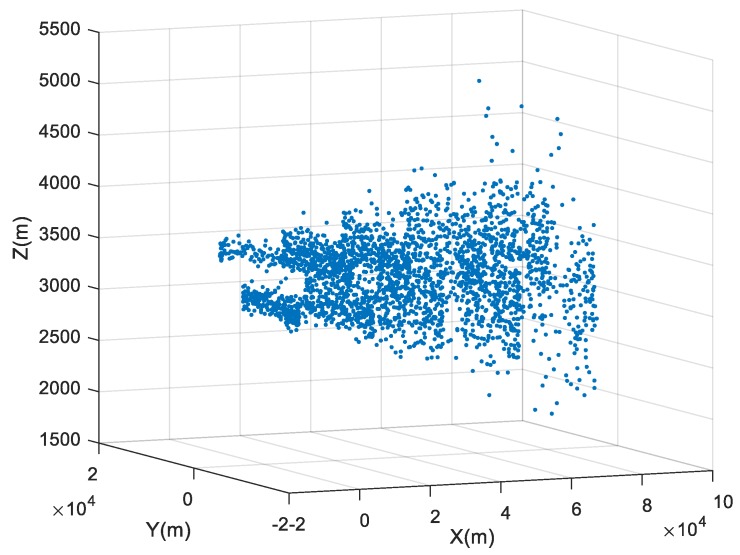
The relative location of two maneuvering targets at each time.

Firstly, the relative location of two maneuvering targets at each time can be obtained with the data in Table 1, which can be shown in Figure 2.

Next, the criterion to decide each track point belonging to which maneuvering target is established. Intuitively, location should be considered from data parameters, including the distance, azimuth angle, and pitch angle. Considering that the recording data correspond to the flight envelopes of two maneuvering targets, there is a limit to the height of the targets. Moreover, speed is also a criterion because two track points for a maneuvering target cannot be far apart. If two points are far apart, one point may be an interference point. Therefore, we mainly take into account three evaluation indices: (1) $C_{1}$: location; (2) $C_{2}$: height; and (3) $C_{3}$: speed. The weight vector of the attributes is $\omega ={\left(0.2,0.4,0.4\right)}^{T}$, and the weight vector of the sensors is $w={\left(1/3,1/3,1/3\right)}^{T}$. Next, we set up the individual evaluation matrix with NPNLTSs by each sensor considering the errors of the sensor. The OLTS and the ILTS are defined as follows:

$$OS=\left\{s_{0}=target\;1,s_{1}=target\;2\right\}$$

$$IS=\left\{n_{0}=dista\;nce\;error,n_{1}=azimuth\;angle\;error,n_{2}=pitch\;angle\;error\right\}.$$

In order to evaluate each track point $T=\left\{t_{1},t_{2},\cdots ,t_{2649}\right\}$, the elements of OLTS are maneuvering target labels, while the elements of ILTS are error parameters. Combined with the systemic error parameters of using sensors shown in Table 2, part of each individual evaluation matrix of each sensor are shown in Table 3, Table 4 and Table 5.

Since the first target point is an initial point, which could be target 1 or target 2, the probabilities for target 1 and target 2 are 0.5 for each attribute. In Table 3, because the evaluation information is collected by sensor 1, the corresponding elements of ILTS are the same, and it also applies to sensor 2 and sensor 3 in Table 4 and Table 5, respectively.

Then, we calculate the collective evaluation matrix based on the associated weight vectors over sensors $w={\left(1/3,1/3,1/3\right)}^{T}$, part of the results is shown in Table 6.

In the following, we aggregate the individual overall evaluation vectors with the weighting vector $\omega ={\left(0.2,0.4,0.4\right)}^{T}$, and part of the results is shown in Table 7.

After that, we aggerate the collective overall evaluation vector with the associated weight vector $\omega ={\left(0.2,0.4,0.4\right)}^{T}$ based on Table 6 and Table 7, part of the results is shown in Table 8.

### 4.3. Solving the Problem

In this subsection, we solve the problem based on the proposed model, and we set the values of the consensus threshold ξ and the adjustment coefficient τ as ξ=0.5 and τ=1/2. According to Equation (7), we calculate the consensus degree $CD\left(s_{1},s_{2},\cdots ,s_{k}\right)=0.78$. Obviously, $CD\left(s_{1},s_{2},\cdots ,s_{k}\right)>0.5$ and we need to adjust the individual evaluation matrix from Table 3 to Table 5 by Equation (8). After 68 times, $CD\left(s_{1},s_{2},\cdots ,s_{k}\right)=0.49$ and it satisfies that $CD\left(s_{1},s_{2},\cdots ,s_{k}\right)\leq 0.5$. At this point, the final group evaluation matrix is obtained, and part of the results is shown in Table 9.

Finally, the final evaluation vector with a consensus can also be calculated based on the associated weight vector, and part of the results is shown in Table 10.

**Figure 3:**
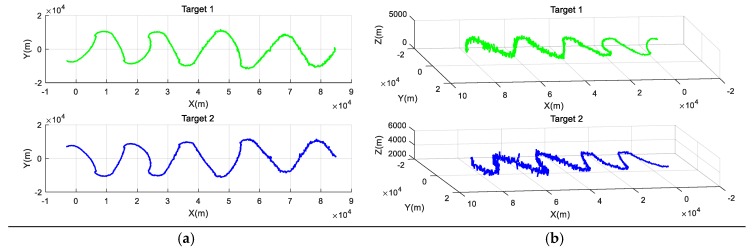
The corresponding tracks of two maneuvering targets. (**a**) The corresponding tracks in two dimensions; (**b**) The corresponding tracks in three dimensions.

Naturally, the corresponding tracks of the two maneuvering targets both in two dimensions and in three dimensions can be shown in Figure 3.

**Figure 4:**
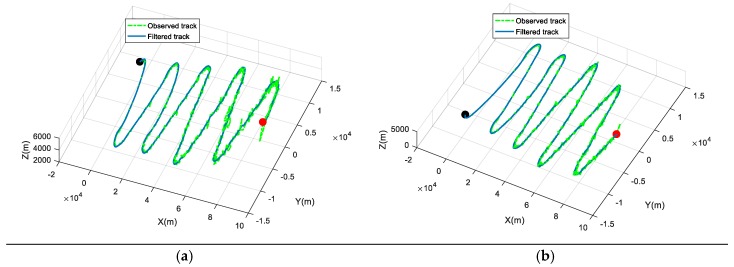
The tracking of two maneuvering targets by Extended Kalman Filter (EKF). (**a**) The tracking of target 1; (**b**) The tracking of target 2.

As we can see, the tracks of the two maneuvering targets are described successfully based on the proposed model. Specifically, there is a total of 1324 track points for target 1, 1310 track points for target 2, and 15 singularities. Additionally, we present the tracking of two maneuvering targets by the Extended Kalman Filter (EKF) algorithm, shown in Figure 4.

In Figure 4, the dotted green line represents the observed track, and the solid blue line represents the filtered track. Moreover, the red point is the starting point and the black point is the end point. The tracks are separated clearly based on the proposed model and the EKF algorithm.

## 5. Comparison and Discussion

In this section, we mainly make further comparisons and analyses to illustrate the effectiveness and applicability of the proposed method and discuss how to deal with the situation when there is only one echo point at a time.

### 5.1. Comparative Analysis

In order to show the superiority of the proposed method compared with other methods, we make analyses from three aspects:

(1) The average root-mean-square error (RMSE) of the key parameters.
(2) The impact of the number of the track points on the average RMSE.
(3) The average operation time (AOT).

At present, there are some popular tracking methods used to deal with track association problems with multiple sensors. For example, several algorithms about tracking problems have been proposed, such as GMM [9], JPDA [12], and MHT [10], and some uncertain linguistic expression methods which can describe track association problems have been put forward, such as HFLTSs [27], PLTSs [28], and DHHFLTSs [29]. Considering both algorithms and expressions, we compare the proposed method with three methods, which are JPDA (method 1), tracking with HFLTSs (method 2), and tracking with PLTSs (method 3).

Firstly, we compare the average RMSE of the key parameters, which are distance, azimuth angle, and pitch angle, among the methods above, and the results are shown in Table 11.

As we can see, the minimum average RMSE of all key parameters is obtained by using the proposed method. The reason may be that method 1 is a single scan processing method, and there is uncertain information when judging each track point. Although method 2 and method 3 can express evaluation information based on cognition, the expression forms cannot contain the errors of key parameters.

Next, varying the number of track points is considered, and the impact of the number of the track points on the average RMSE among four methods is shown in Table 12.

In Table 12, the results indicate that the proposed method is very effective in terms of average RMSE of all the key parameters. Additionally, it is shown that increasing the number of track points can improve tracking performance no matter what method is used. However, as the number of track points increases, it is also observed that the performance improvement dwindles gradually.

Finally, since the AOT is a key factor to evaluate the method, we compare the AOT with various numbers of track points among four methods. Specifically, we can obtain the AOT of different methods by using the functions “tic” and “toc” in MATLAB. And the result is shown in Table 13.

Table 13 indicates that the AOT requires various numbers of track points for each method. In the case of three situations, the proposed method requires the minimum time among the four methods. Moreover, as the number of track points increases, it is observed that AOT increases for all methods. Therefore, the proposed method is superior to other three methods in terms of the three aspects above.

### 5.2. Discussion

During the practical process of track association, abnormal track points usually appear when there is only one echo point at a time. In order not to lose the track, based on a filter technique, we can make discriminant analysis performed by using the correlation between the time before and after the target point.

Firstly, we need to convert the measured parameters (d,α,β) to rectangular coordinate Z(k), and predict the predicted value of target measurement at the current moment by the sensor observation equation:

(9) Z(k|k−1)=H(k)X(k|k−1).

where H(k) is a measurement matrix, and is expressed as:

(10) $$H\left(k\right)=\left[\begin{array}{cccccc} 1 & 0 & 0 & 0 & 0 & 0\\ 0 & 0 & 1 & 0 & 0 & 0\\ 0 & 0 & 0 & 0 & 1 & 0 \end{array}\right].$$

X(k|k−1) is the predicted state value at the current moment in the rectangular coordinate, and the expression is $X\left(k|k-1\right)=\left[\begin{array}{cccccc} x & x & y & y & z & z \end{array}\right]$.

Obviously, when there is an abnormal track point, the residual can increase. Next, we define the expression of the residual variable:

(11) v(k)=Z(k)−Z(k|k−1).

where v(k) is a random variable of Gaussian distribution with mean 0, and its covariance is:

(12) $$E\left[v\left(k\right)v{\left(k\right)}^{T}\right]=H\left(k\right)P\left(k|k-1\right)H^{T}\left(k\right)+R\left(k\right).$$

where P(k|k−1) is the predicted covariance, and R(k) is the measurement noise covariance.

In the following, we can distinguish each component in the measured value according to the residual of the track point and its statistical properties, and the discriminant is:

(13) $$\left|v_{i}\left(k\right)\right|\leq C\sqrt{{\left[H\left(k\right)P\left(k|k-1\right)H^{T}\left(k\right)+R\left(k\right)\right]}_{i.i}}+L_{i}.$$

where i represents the *i*-th element on the diagonal of the matrix, $v_{i}\left(k\right)$ is the *i*-th component in v(k), $L_{i}$ is the length of a target point in one direction, $H^{T}$ is the transpose of H, and C is a constant decided by an actual situation, and set C=3 or C=4 in general.

If each component in the residual of the track point satisfies Equation (13), then this point is the observation point; otherwise, it is an abnormal point. Therefore, we can distinguish situations where there is only one echo point at a time. Additionally, the ability of the residual to recognize abnormal points depends on the prediction accuracy and the value $E\left[v\left(k\right)v{\left(k\right)}^{T}\right]$. The higher the prediction accuracy, the stronger the ability to distinguish abnormal points.

## 6. Conclusions

Target tracking is an important technology in many fields, such as military, transportation, and navigation. Especially, when there are multiple targets in flight at the same time, precisely distinguishing the corresponding track is a key problem. In this paper, a consensus-based track association method is proposed with multistatic sensors under a nested probabilistic-numerical linguistic environment. The proposed method seeks to solve the problem of multiple target tracking by following two procedures. Firstly, sensors need to express uncertain information about the targets with NPNLTSs, because it can contain errors of key parameters and describe information more accurately. Second, the consensus model with NPNLTSs has been put forward. The model involves a consensus checking process and a consensus modifying process, and it can improve the consensus greatly among sensors during this process. According to the case study, it is observed that the proposed method is capable of track association of multiple targets with multistatic sensors. Additionally, in the comparisons with three other popular methods, the performance of the proposed method shows superior effectiveness and applicability in terms of both accuracy and computational complexity. Moreover, we have discussed the situation where there is only one echo point at a time, and have given the discriminant analysis method in detail.

There are some interesting topics for further research. For example, new frontiers and challenges can be established in the fields of consensus reaching models for dynamic environments, intelligent negotiation processes, and AI algorithms that improve decision making in sensor networks. Furthermore, we can apply more accurate data using machine learning algorithms according to case studies or state-of-the-art reviews.

## Figures and Tables

**Table 1:** Part of the measurement data of two targets measured with three sensors.

Distance (m)	Azimuth Angle (degree)	Pitch Angle (degree)	Time (s)	Sensor Label
84,626.83	89.99	1.74	0.10	1
85,016.58	89.50	3.07	0.70	1
…	…	…	…	1
53,481.28	87.24	3.93	272.50	1
48,556.38	233.38	3.02	272.60	2
48,538.89	227.05	3.50	273.10	2
…	…	…	…	2
20,360.67	248.65	7.49	542.50	2
25,166.65	331.88	8.21	543.00	3
25,229.30	−8.92	9.50	543.10	3
…	…	…	…	3
32,031.73	−104.41	29.11	808.90	3

**Table 2:** System information of the three sensors.

Sensor Label	Longitude ω (degree)	Latitude ϕ (degree)	Height h (m)	Error of D (m)	Error of α (degree)	Error of β (degree)
1	102.1	30.5	0	50	0.4	0.4
2	102.4	31.5	0	60	0.5	0.5
3	102.7	31.9	0	60	0.5	0.5

**Table 3:** Part of the individual evaluation matrix of sensor 1.

Sensor 1	Location	Height	Speed
$t_{1}$	$\left\{\begin{array}{l} s_{0}(0.5)\left\{n_{0}\left(50\right),n_{1}\left(0.4\right),n_{2}\left(0.4\right)\right\},\\ s_{1}(0.5)\left\{n_{0}\left(50\right),n_{1}\left(0.4\right),n_{2}\left(0.4\right)\right\} \end{array}\right\}$	$\left\{\begin{array}{l} s_{0}(0.5)\left\{n_{0}\left(50\right),n_{1}\left(0.4\right),n_{2}\left(0.4\right)\right\},\\ s_{1}(0.5)\left\{n_{0}\left(50\right),n_{1}\left(0.4\right),n_{2}\left(0.4\right)\right\} \end{array}\right\}$	$\left\{\begin{array}{l} s_{0}(0.5)\left\{n_{0}\left(50\right),n_{1}\left(0.4\right),n_{2}\left(0.4\right)\right\},\\ s_{1}(0.5)\left\{n_{0}\left(50\right),n_{1}\left(0.4\right),n_{2}\left(0.4\right)\right\} \end{array}\right\}$
$t_{2}$	$\left\{\begin{array}{l} s_{0}(0.5)\left\{n_{0}\left(50\right),n_{1}\left(0.4\right),n_{2}\left(0.4\right)\right\},\\ s_{1}(0.5)\left\{n_{0}\left(50\right),n_{1}\left(0.4\right),n_{2}\left(0.4\right)\right\} \end{array}\right\}$	$\left\{\begin{array}{l} s_{0}(0.8)\left\{n_{0}\left(50\right),n_{1}\left(0.4\right),n_{2}\left(0.4\right)\right\},\\ s_{1}(0.2)\left\{n_{0}\left(50\right),n_{1}\left(0.4\right),n_{2}\left(0.4\right)\right\} \end{array}\right\}$	$\left\{\begin{array}{l} s_{0}(0.7)\left\{n_{0}\left(50\right),n_{1}\left(0.4\right),n_{2}\left(0.4\right)\right\},\\ s_{1}(0.3)\left\{n_{0}\left(50\right),n_{1}\left(0.4\right),n_{2}\left(0.4\right)\right\} \end{array}\right\}$
…	…	…	…
$t_{2649}$	$\left\{\begin{array}{l} s_{0}(0.2)\left\{n_{0}\left(50\right),n_{1}\left(0.4\right),n_{2}\left(0.4\right)\right\},\\ s_{1}(0.8)\left\{n_{0}\left(50\right),n_{1}\left(0.4\right),n_{2}\left(0.4\right)\right\} \end{array}\right\}$	$\left\{\begin{array}{l} s_{0}(0.4)\left\{n_{0}\left(50\right),n_{1}\left(0.4\right),n_{2}\left(0.4\right)\right\},\\ s_{1}(0.6)\left\{n_{0}\left(50\right),n_{1}\left(0.4\right),n_{2}\left(0.4\right)\right\} \end{array}\right\}$	$\left\{\begin{array}{l} s_{0}(0.3)\left\{n_{0}\left(50\right),n_{1}\left(0.4\right),n_{2}\left(0.4\right)\right\},\\ s_{1}(0.7)\left\{n_{0}\left(50\right),n_{1}\left(0.4\right),n_{2}\left(0.4\right)\right\} \end{array}\right\}$

**Table 4:** Part of the individual evaluation matrix of sensor 2.

Sensor 2	Location	Height	Speed
$t_{1}$	$\left\{\begin{array}{l} s_{0}(0.3)\left\{n_{0}\left(60\right),n_{1}\left(0.5\right),n_{2}\left(0.5\right)\right\},\\ s_{1}(0.7)\left\{n_{0}\left(60\right),n_{1}\left(0.5\right),n_{2}\left(0.5\right)\right\} \end{array}\right\}$	$\left\{\begin{array}{l} s_{0}(0.3)\left\{n_{0}\left(60\right),n_{1}\left(0.5\right),n_{2}\left(0.5\right)\right\},\\ s_{1}(0.7)\left\{n_{0}\left(60\right),n_{1}\left(0.5\right),n_{2}\left(0.5\right)\right\} \end{array}\right\}$	$\left\{\begin{array}{l} s_{0}(0.4)\left\{n_{0}\left(60\right),n_{1}\left(0.5\right),n_{2}\left(0.5\right)\right\},\\ s_{1}(0.6)\left\{n_{0}\left(60\right),n_{1}\left(0.5\right),n_{2}\left(0.5\right)\right\} \end{array}\right\}$
$t_{2}$	$\left\{\begin{array}{l} s_{0}(0.6)\left\{n_{0}\left(60\right),n_{1}\left(0.5\right),n_{2}\left(0.5\right)\right\},\\ s_{1}(0.4)\left\{n_{0}\left(60\right),n_{1}\left(0.5\right),n_{2}\left(0.5\right)\right\} \end{array}\right\}$	$\left\{\begin{array}{l} s_{0}(0.7)\left\{n_{0}\left(60\right),n_{1}\left(0.5\right),n_{2}\left(0.5\right)\right\},\\ s_{1}(0.3)\left\{n_{0}\left(60\right),n_{1}\left(0.5\right),n_{2}\left(0.5\right)\right\} \end{array}\right\}$	$\left\{\begin{array}{l} s_{0}(0.7)\left\{n_{0}\left(60\right),n_{1}\left(0.5\right),n_{2}\left(0.5\right)\right\},\\ s_{1}(0.3)\left\{n_{0}\left(60\right),n_{1}\left(0.5\right),n_{2}\left(0.5\right)\right\} \end{array}\right\}$
…	…	…	…
$t_{2649}$	$\left\{\begin{array}{l} s_{0}(0.8)\left\{n_{0}\left(60\right),n_{1}\left(0.5\right),n_{2}\left(0.5\right)\right\},\\ s_{1}(0.2)\left\{n_{0}\left(60\right),n_{1}\left(0.5\right),n_{2}\left(0.5\right)\right\} \end{array}\right\}$	$\left\{\begin{array}{l} s_{0}(0.6)\left\{n_{0}\left(60\right),n_{1}\left(0.5\right),n_{2}\left(0.5\right)\right\},\\ s_{1}(0.4)\left\{n_{0}\left(60\right),n_{1}\left(0.5\right),n_{2}\left(0.5\right)\right\} \end{array}\right\}$	$\left\{\begin{array}{l} s_{0}(0.8)\left\{n_{0}\left(60\right),n_{1}\left(0.5\right),n_{2}\left(0.5\right)\right\},\\ s_{1}(0.2)\left\{n_{0}\left(60\right),n_{1}\left(0.5\right),n_{2}\left(0.5\right)\right\} \end{array}\right\}$

**Table 5:** Part of the individual evaluation matrix of sensor 3.

Sensor 3	Location	Height	Speed
$t_{1}$	$\left\{\begin{array}{l} s_{0}(0.5)\left\{n_{0}\left(60\right),n_{1}\left(0.5\right),n_{2}\left(0.5\right)\right\},\\ s_{1}(0.5)\left\{n_{0}\left(60\right),n_{1}\left(0.5\right),n_{2}\left(0.5\right)\right\} \end{array}\right\}$	$\left\{\begin{array}{l} s_{0}(0.4)\left\{n_{0}\left(60\right),n_{1}\left(0.5\right),n_{2}\left(0.5\right)\right\},\\ s_{1}(0.6)\left\{n_{0}\left(60\right),n_{1}\left(0.5\right),n_{2}\left(0.5\right)\right\} \end{array}\right\}$	$\left\{\begin{array}{l} s_{0}(0.3)\left\{n_{0}\left(60\right),n_{1}\left(0.5\right),n_{2}\left(0.5\right)\right\},\\ s_{1}(0.7)\left\{n_{0}\left(60\right),n_{1}\left(0.5\right),n_{2}\left(0.5\right)\right\} \end{array}\right\}$
$t_{2}$	$\left\{\begin{array}{l} s_{0}(0.6)\left\{n_{0}\left(60\right),n_{1}\left(0.5\right),n_{2}\left(0.5\right)\right\},\\ s_{1}(0.4)\left\{n_{0}\left(60\right),n_{1}\left(0.5\right),n_{2}\left(0.5\right)\right\} \end{array}\right\}$	$\left\{\begin{array}{l} s_{0}(0.5)\left\{n_{0}\left(60\right),n_{1}\left(0.5\right),n_{2}\left(0.5\right)\right\},\\ s_{1}(0.5)\left\{n_{0}\left(60\right),n_{1}\left(0.5\right),n_{2}\left(0.5\right)\right\} \end{array}\right\}$	$\left\{\begin{array}{l} s_{0}(0.7)\left\{n_{0}\left(60\right),n_{1}\left(0.5\right),n_{2}\left(0.5\right)\right\},\\ s_{1}(0.3)\left\{n_{0}\left(60\right),n_{1}\left(0.5\right),n_{2}\left(0.5\right)\right\} \end{array}\right\}$
…	…	…	…
$t_{2649}$	$\left\{\begin{array}{l} s_{0}(0.6)\left\{n_{0}\left(60\right),n_{1}\left(0.5\right),n_{2}\left(0.5\right)\right\},\\ s_{1}(0.4)\left\{n_{0}\left(60\right),n_{1}\left(0.5\right),n_{2}\left(0.5\right)\right\} \end{array}\right\}$	$\left\{\begin{array}{l} s_{0}(0.7)\left\{n_{0}\left(60\right),n_{1}\left(0.5\right),n_{2}\left(0.5\right)\right\},\\ s_{1}(0.3)\left\{n_{0}\left(60\right),n_{1}\left(0.5\right),n_{2}\left(0.5\right)\right\} \end{array}\right\}$	$\left\{\begin{array}{l} s_{0}(0.5)\left\{n_{0}\left(60\right),n_{1}\left(0.5\right),n_{2}\left(0.5\right)\right\},\\ s_{1}(0.5)\left\{n_{0}\left(60\right),n_{1}\left(0.5\right),n_{2}\left(0.5\right)\right\} \end{array}\right\}$

**Table 6:** Part of the collective evaluation matrix.

	Location	Height	Speed
$t_{1}$	$\left\{\begin{array}{l} s_{0}(0.43)\left\{n_{0}\left(57\right),n_{1}\left(0.47\right),n_{2}\left(0.47\right)\right\},\\ s_{1}(0.57)\left\{n_{0}\left(57\right),n_{1}\left(0.47\right),n_{2}\left(0.47\right)\right\} \end{array}\right\}$	$\left\{\begin{array}{l} s_{0}(0.40)\left\{n_{0}\left(57\right),n_{1}\left(0.47\right),n_{2}\left(0.47\right)\right\},\\ s_{1}(0.60)\left\{n_{0}\left(57\right),n_{1}\left(0.47\right),n_{2}\left(0.47\right)\right\} \end{array}\right\}$	$\left\{\begin{array}{l} s_{0}(0.40)\left\{n_{0}\left(57\right),n_{1}\left(0.47\right),n_{2}\left(0.47\right)\right\},\\ s_{1}(0.60)\left\{n_{0}\left(57\right),n_{1}\left(0.47\right),n_{2}\left(0.47\right)\right\} \end{array}\right\}$
$t_{2}$	$\left\{\begin{array}{l} s_{0}(0.57)\left\{n_{0}\left(57\right),n_{1}\left(0.47\right),n_{2}\left(0.47\right)\right\},\\ s_{1}(0.43)\left\{n_{0}\left(57\right),n_{1}\left(0.47\right),n_{2}\left(0.47\right)\right\} \end{array}\right\}$	$\left\{\begin{array}{l} s_{0}(0.67)\left\{n_{0}\left(57\right),n_{1}\left(0.47\right),n_{2}\left(0.47\right)\right\},\\ s_{1}(0.33)\left\{n_{0}\left(57\right),n_{1}\left(0.47\right),n_{2}\left(0.47\right)\right\} \end{array}\right\}$	$\left\{\begin{array}{l} s_{0}(0.70)\left\{n_{0}\left(57\right),n_{1}\left(0.47\right),n_{2}\left(0.47\right)\right\},\\ s_{1}(0.30)\left\{n_{0}\left(57\right),n_{1}\left(0.47\right),n_{2}\left(0.47\right)\right\} \end{array}\right\}$
…	…	…	…
$t_{2649}$	$\left\{\begin{array}{l} s_{0}(0.53)\left\{n_{0}\left(57\right),n_{1}\left(0.47\right),n_{2}\left(0.47\right)\right\},\\ s_{1}(0.47)\left\{n_{0}\left(57\right),n_{1}\left(0.47\right),n_{2}\left(0.47\right)\right\} \end{array}\right\}$	$\left\{\begin{array}{l} s_{0}(0.57)\left\{n_{0}\left(57\right),n_{1}\left(0.47\right),n_{2}\left(0.47\right)\right\},\\ s_{1}(0.43)\left\{n_{0}\left(57\right),n_{1}\left(0.47\right),n_{2}\left(0.47\right)\right\} \end{array}\right\}$	$\left\{\begin{array}{l} s_{0}(0.53)\left\{n_{0}\left(57\right),n_{1}\left(0.47\right),n_{2}\left(0.47\right)\right\},\\ s_{1}(0.47)\left\{n_{0}\left(57\right),n_{1}\left(0.47\right),n_{2}\left(0.47\right)\right\} \end{array}\right\}$

**Table 7:** Part of the individual overall evaluation vectors.

	Sensor 1	Sensor 2	Sensor 3
$t_{1}$	$\left\{\begin{array}{l} s_{0}(0.50)\left\{n_{0}\left(50\right),n_{1}\left(0.4\right),n_{2}\left(0.4\right)\right\},\\ s_{1}(0.50)\left\{n_{0}\left(50\right),n_{1}\left(0.4\right),n_{2}\left(0.4\right)\right\} \end{array}\right\}$	$\left\{\begin{array}{l} s_{0}(0.34)\left\{n_{0}\left(60\right),n_{1}\left(0.5\right),n_{2}\left(0.5\right)\right\},\\ s_{1}(0.66)\left\{n_{0}\left(60\right),n_{1}\left(0.5\right),n_{2}\left(0.5\right)\right\} \end{array}\right\}$	$\left\{\begin{array}{l} s_{0}(0.38)\left\{n_{0}\left(60\right),n_{1}\left(0.5\right),n_{2}\left(0.5\right)\right\},\\ s_{1}(0.62)\left\{n_{0}\left(60\right),n_{1}\left(0.5\right),n_{2}\left(0.5\right)\right\} \end{array}\right\}$
$t_{2}$	$\left\{\begin{array}{l} s_{0}(0.70)\left\{n_{0}\left(50\right),n_{1}\left(0.4\right),n_{2}\left(0.4\right)\right\},\\ s_{1}(0.30)\left\{n_{0}\left(50\right),n_{1}\left(0.4\right),n_{2}\left(0.4\right)\right\} \end{array}\right\}$	$\left\{\begin{array}{l} s_{0}(0.68)\left\{n_{0}\left(60\right),n_{1}\left(0.5\right),n_{2}\left(0.5\right)\right\},\\ s_{1}(0.32)\left\{n_{0}\left(60\right),n_{1}\left(0.5\right),n_{2}\left(0.5\right)\right\} \end{array}\right\}$	$\left\{\begin{array}{l} s_{0}(0.60)\left\{n_{0}\left(60\right),n_{1}\left(0.5\right),n_{2}\left(0.5\right)\right\},\\ s_{1}(0.40)\left\{n_{0}\left(60\right),n_{1}\left(0.5\right),n_{2}\left(0.5\right)\right\} \end{array}\right\}$
…	…	…	…
$t_{2649}$	$\left\{\begin{array}{l} s_{0}(0.32)\left\{n_{0}\left(50\right),n_{1}\left(0.4\right),n_{2}\left(0.4\right)\right\},\\ s_{1}(0.68)\left\{n_{0}\left(50\right),n_{1}\left(0.4\right),n_{2}\left(0.4\right)\right\} \end{array}\right\}$	$\left\{\begin{array}{l} s_{0}(0.72)\left\{n_{0}\left(60\right),n_{1}\left(0.5\right),n_{2}\left(0.5\right)\right\},\\ s_{1}(0.28)\left\{n_{0}\left(60\right),n_{1}\left(0.5\right),n_{2}\left(0.5\right)\right\} \end{array}\right\}$	$\left\{\begin{array}{l} s_{0}(0.60)\left\{n_{0}\left(60\right),n_{1}\left(0.5\right),n_{2}\left(0.5\right)\right\},\\ s_{1}(0.40)\left\{n_{0}\left(60\right),n_{1}\left(0.5\right),n_{2}\left(0.5\right)\right\} \end{array}\right\}$

**Table 8:** Part of the collective overall evaluation vectors.

$t_{1}$	$\left\{s_{0}(0.406)\left\{n_{0}\left(57\right),n_{1}\left(0.47\right),n_{2}\left(0.47\right)\right\},s_{1}(0.594)\left\{n_{0}\left(57\right),n_{1}\left(0.47\right),n_{2}\left(0.47\right)\right\}\right\}$
$t_{2}$	$\left\{s_{0}(0.662)\left\{n_{0}\left(57\right),n_{1}\left(0.47\right),n_{2}\left(0.47\right)\right\},s_{1}(0.338)\left\{n_{0}\left(57\right),n_{1}\left(0.47\right),n_{2}\left(0.47\right)\right\}\right\}$
…	…
$t_{2649}$	$\left\{s_{0}(0.546)\left\{n_{0}\left(57\right),n_{1}\left(0.47\right),n_{2}\left(0.47\right)\right\},s_{1}(0.454)\left\{n_{0}\left(57\right),n_{1}\left(0.47\right),n_{2}\left(0.47\right)\right\}\right\}$

**Table 9:** Part of the final group evaluation matrix.

	Location	Height	Speed
$t_{1}$	$\left\{\begin{array}{l} s_{0}(0.35)\left\{n_{0}\left(57\right),n_{1}\left(0.47\right),n_{2}\left(0.47\right)\right\},\\ s_{1}(0.65)\left\{n_{0}\left(57\right),n_{1}\left(0.47\right),n_{2}\left(0.47\right)\right\} \end{array}\right\}$	$\left\{\begin{array}{l} s_{0}(0.30)\left\{n_{0}\left(57\right),n_{1}\left(0.47\right),n_{2}\left(0.47\right)\right\},\\ s_{1}(0.70)\left\{n_{0}\left(57\right),n_{1}\left(0.47\right),n_{2}\left(0.47\right)\right\} \end{array}\right\}$	$\left\{\begin{array}{l} s_{0}(0.28)\left\{n_{0}\left(57\right),n_{1}\left(0.47\right),n_{2}\left(0.47\right)\right\},\\ s_{1}(0.72)\left\{n_{0}\left(57\right),n_{1}\left(0.47\right),n_{2}\left(0.47\right)\right\} \end{array}\right\}$
$t_{2}$	$\left\{\begin{array}{l} s_{0}(0.60)\left\{n_{0}\left(57\right),n_{1}\left(0.47\right),n_{2}\left(0.47\right)\right\},\\ s_{1}(0.40)\left\{n_{0}\left(57\right),n_{1}\left(0.47\right),n_{2}\left(0.47\right)\right\} \end{array}\right\}$	$\left\{\begin{array}{l} s_{0}(0.71)\left\{n_{0}\left(57\right),n_{1}\left(0.47\right),n_{2}\left(0.47\right)\right\},\\ s_{1}(0.29)\left\{n_{0}\left(57\right),n_{1}\left(0.47\right),n_{2}\left(0.47\right)\right\} \end{array}\right\}$	$\left\{\begin{array}{l} s_{0}(0.80)\left\{n_{0}\left(57\right),n_{1}\left(0.47\right),n_{2}\left(0.47\right)\right\},\\ s_{1}(0.20)\left\{n_{0}\left(57\right),n_{1}\left(0.47\right),n_{2}\left(0.47\right)\right\} \end{array}\right\}$
…	…	…	…
$t_{2649}$	$\left\{\begin{array}{l} s_{0}(0.58)\left\{n_{0}\left(57\right),n_{1}\left(0.47\right),n_{2}\left(0.47\right)\right\},\\ s_{1}(0.42)\left\{n_{0}\left(57\right),n_{1}\left(0.47\right),n_{2}\left(0.47\right)\right\} \end{array}\right\}$	$\left\{\begin{array}{l} s_{0}(0.67)\left\{n_{0}\left(57\right),n_{1}\left(0.47\right),n_{2}\left(0.47\right)\right\},\\ s_{1}(0.33)\left\{n_{0}\left(57\right),n_{1}\left(0.47\right),n_{2}\left(0.47\right)\right\} \end{array}\right\}$	$\left\{\begin{array}{l} s_{0}(0.68)\left\{n_{0}\left(57\right),n_{1}\left(0.47\right),n_{2}\left(0.47\right)\right\},\\ s_{1}(0.32)\left\{n_{0}\left(57\right),n_{1}\left(0.47\right),n_{2}\left(0.47\right)\right\} \end{array}\right\}$

**Table 10:** Part of the final evaluation vector with a consensus.

$t_{1}$	$\left\{s_{0}(0.302)\left\{n_{0}\left(57\right),n_{1}\left(0.47\right),n_{2}\left(0.47\right)\right\},s_{1}(0.698)\left\{n_{0}\left(57\right),n_{1}\left(0.47\right),n_{2}\left(0.47\right)\right\}\right\}$
$t_{2}$	$\left\{s_{0}(0.724)\left\{n_{0}\left(57\right),n_{1}\left(0.47\right),n_{2}\left(0.47\right)\right\},s_{1}(0.276)\left\{n_{0}\left(57\right),n_{1}\left(0.47\right),n_{2}\left(0.47\right)\right\}\right\}$
…	…
$t_{2649}$	$\left\{s_{0}(0.656)\left\{n_{0}\left(57\right),n_{1}\left(0.47\right),n_{2}\left(0.47\right)\right\},s_{1}(0.344)\left\{n_{0}\left(57\right),n_{1}\left(0.47\right),n_{2}\left(0.47\right)\right\}\right\}$

**Table 11:** The average root-mean square error (RMSE) of key parameters among four methods.

Average RMSE	Method 1 [12]	Method 2 [27]	Method 3 [28]	Proposed Method
Distance (m)	66.93	63.21	57.32	52.12
Azimuth angle (degree)	0.60	0.55	0.52	0.42
Pitch angle (degree)	0.58	0.52	0.50	0.41

**Table 12:** The average RMSE for varying the number of track points among four methods.

Average RMSE	Number	Method 1 [12]	Method 2 [27]	Method 3 [28]	Proposed Method
Distance (m)	1000	75.73	74.25	64.72	59.13
	2000	66.94	65.98	58.25	53.72
	3000	64.78	62.56	56.29	50.44
Azimuth angle (degree)	1000	0.63	0.59	0.57	0.46
	2000	0.60	0.55	0.52	0.42
	3000	0.58	0.54	0.50	0.40
Pitch angle (degree)	1000	0.63	0.55	0.53	0.45
	2000	0.59	0.52	0.50	0.41
	3000	0.58	0.50	0.48	0.40

**Table 13:** The average operation time (AOT) with various number of track points among four methods.

Number	AOT (s)			
	Method 1 [12]	Method 2 [27]	Method 3 [28]	Proposed Method
1000	2.67	2.03	1.67	0.65
2000	3.23	2.98	2.67	1.12
3000	5.87	5.03	4.78	2.67

## References

[1] Attari M., Habibi S., Gadsden S.A. (2017). Target tracking formulation of the SVSF with data association techniques. IEEE Trans. Aerosp. Electron. Syst..

[2] Zhu S.H., Shi Z., Sun C.J. (2016). Tracklet association based multi-target tracking. Multimedia Tools Appl..

[3] Hu X.Q., Bao M., Zhang X.P., Wen S., Li X.D., Hu Y.H. (2018). Quantized kalman filter tracking in directional sensor networks. IEEE Trans. Mob. Comput..

[4] Fortino G., Galzarano S., Gravina R., Li W.F. (2015). A framework for collaborative computing and multisensor data fusion in body sensor networks. Inf. Fusion.

[5] Gravina R., Alinia P., Ghassemzadeh H., Fortino G. (2017). Multisensor fusion in body sensor networks: State-of-the-art and research challenges. Inf. Fusion.

[6] Kazimierski W. (2017). Proposal of neural approach to maritime radar and automatic identification system tracks association. IET Radar Sonar Navig..

[7] Yoon J.H., Lee C.R., Yang M.H., Yoon K.J. (2019). Structural constraint data association for online multi-object tracking. Int. J. Comput. Vis..

[8] Yang M., Wu Y.W., Jia Y.D. (2017). A hybrid data association framework for robust online multi-object tracking. IEEE Trans. Image Process..

[9] Ma J.Y., Jiang J.J., Liu C.Y., Li Y.S. (2017). Feature guided Gaussian mixture model with semi-supervised EM and local geometric constraint for retinal image registration. Inf. Sci..

[10] Coraluppi S.P., Carthel C.A. (2018). Multiple-hypothesis tracking for targets producing multiple measurements. IEEE Trans. Aerosp. Electron. Syst..

[11] Chen X., Li Y.A., Li Y.X., Yu J., Li X.H. (2016). A novel probabilistic data association for target tracking in a cluttered environment. Sensors.

[12] Vivone G., Braca P. (2016). Joint probabilistic data association tracker for extended target tracking applied to X-band marine radar data. IEEE J. Ocean. Eng..

[13] Fan L.X., Fan E., Yuan C.H., Hu K.L. (2016). Weighted fuzzy track association method based on Dempster-Shafer theory in distributed sensor networks. Int. J. Distrib. Sens. Netw..

[14] Li J., Xie W.X., Li L.Q. (2017). Online visual multiple target tracking by intuitionistic fuzzy data association. Int. J. Fuzzy Syst..

[15] Yang D., Ji H.B., Gao Y.C. (2019). A robust D-S fusion algorithm for multi-target multisensor with higher reliability. Inf. Fusion.

[16] Yoon K., Kim D.Y., Yoon Y.C., Jeon M. (2019). Data association for multi-object tracking via deep neural networks. Sensors.

[17] Scott S.L., Blocker A.W., Bonassi F.V., Chipman H.A., George E.I., McCulloch R.E. (2016). Bayes and big data: The consensus Monte Carlo algorithm. Int. J. Eng. Sci..

[18] Luengo D., Martino L., Elvira V., Bugallo M.F. (2018). Efficient linear fusion of partial estimators. Digit. Signal Process..

[19] Olfati-Saber R., Fax J.A., Murray R.M. (2007). Consensus and cooperation in networked multi-agent systems. Proc. IEEE.

[20] Dimakis A.G., Kar S., Moura J.F., Rabbat M.G., Scaglione A. (2010). Gossip algorithms for distributed signal processing. Proc. IEEE.

[21] Yu Y.H. (2017). Distributed target tracking in wireless sensor networks with data association uncertainty. IEEE Commun. Lett..

[22] Wang X.X., Xu Z.S., Gou X.J. (2019). Nested probabilistic-numerical linguistic term sets in two-stage multi-attribute group decision making. Appl. Intell..

[23] Wang X.X., Xu Z.S., Gou X.J., Trajkovic L. (2019). Tracking a maneuvering target by multiple sensors using extended kalman filter with nested probabilistic-numerical linguistic information. IEEE Trans. Fuzzy Syst..

[24] Liao H.C., Xu Z.S., Zeng X.J., Merigo J.M. (2015). Qualitative decision making with correlation coefficients of hesitant fuzzy linguistic term sets. Knowl.-Based Syst..

[25] Wei C.P., Zhao N., Tang X.J. (2014). Operators and comparisons of hesitant fuzzy linguistic term sets. IEEE Trans. Fuzzy Syst..

[26] Zhang Y.X., Xu Z.S., Liao H.C. (2017). A consensus process for group decision making with probabilistic linguistic preference relations. Inf. Sci..

[27] Rodriguez R.M., Martinez L., Herrera F. (2012). Hesitant fuzzy linguistic term sets for decision making. IEEE Trans. Fuzzy Syst..

[28] Pang Q., Wang H., Xu Z.S. (2016). Probabilistic linguistic term sets in multi-attribute group decision making. Inf. Sci..

[29] Gou X.J., Liao H.C., Xu Z.S., Herrera F. (2017). Double hierarchy hesitant fuzzy linguistic term set and MULTIMOORA method: A case of study to evaluate the implementation status of haze controlling measures. Inf. Fusion.

[30] Wang X.X., Xu Z.S., Gou X.J. (2019). Distance and similarity measures for nested probabilistic-numerical linguistic term sets applied to evaluation of medical treatment. Int. J. Fuzzy Syst..

[31] Karplus P.A., Diederichs K. (2012). Linking crystallographic model and data quality. Science.

[32] Chiclana F., Tapia Garcia J.M., del Moral M.J., Herrera-Viedma E. (2013). A statistical comparative study of different similarity measures of consensus in group decision making. Inf. Sci..

[33] Herrera-Viedma E., Herrera F., Chiclana F. (2002). A consensus model for multiperson decision making with different preference structures. IEEE Trans. Syst. Man Cybern. Part A Syst. Hum..

[34] Herrera-Viedma E., Martinez L., Mata F., Chiclana F. (2005). A consensus support system model for group decision-making problems with multigranular linguistic preference relations. IEEE Trans. Fuzzy Syst..

[35] Dong Q.X., Cooper O. (2016). A peer-to-peer dynamic adaptive consensus reaching model for the group AHP decision making. Eur. J. Oper. Res..

[36] Ma L.C. (2016). A new group ranking approach for ordinal preferences based on group maximum consensus sequences. Eur. J. Oper. Res..

